# Plasma Treatment Maintains Surface Energy of the Implant Surface and Enhances Osseointegration

**DOI:** 10.1155/2013/354125

**Published:** 2013-01-10

**Authors:** Fernando P. S. Guastaldi, Daniel Yoo, Charles Marin, Ryo Jimbo, Nick Tovar, Darceny Zanetta-Barbosa, Paulo G. Coelho

**Affiliations:** ^1^Department of Biomaterials and Biomimetics, College of Dentistry, New York University, Room 813a, 345 East 24th Street, New York, NY 10010, USA; ^2^Department of Surgery and Integrated Clinic, São Paulo State University, 16015 Araçatuba, SP, Brazil; ^3^Department of Postgraduate Dentistry, UNIGRANRIO, 25071 Duque de Caxias, RJ, Brazil; ^4^Department of Prosthodontics, Faculty of Odontology, Malmö University, 205 06 Malmö, Sweden; ^5^Department of Oral & Maxillofacial Surgery and Implantology, University of Uberlândia, 38408 Uberlândia, MG, Brazil

## Abstract

The surface energy of the implant surface has an impact on osseointegration. In this study, 2 surfaces: nonwashed resorbable blasting media (NWRBM; control) and Ar-based nonthermal plasma 30 days (Plasma 30 days; experimental), were investigated with a focus on the surface energy. The surface energy was characterized by the Owens-Wendt-Rabel-Kaelble method and the chemistry by X-ray photoelectron spectroscopy (XPS). Five adult beagle dogs received 8 implants (*n* = 2 per surface, per tibia). After 2 weeks, the animals were euthanized, and half of the implants (*n* = 20) were removal torqued and the other half were histologically processed (*n* = 20). The bone-to-implant contact (BIC) and bone area fraction occupancy (BAFO) were evaluated on the histologic sections. The XPS analysis showed peaks of C, Ca, O, and P for the control and experimental surfaces. While no significant difference was observed for BIC parameter (*P* > 0.75), a higher level for torque (*P* < 0.02) and BAFO parameter (*P* < 0.01) was observed for the experimental group. The surface elemental chemistry was modified by the plasma and lasted for 30 days after treatment resulting in improved biomechanical fixation and bone formation at 2 weeks compared to the control group.

## 1. Introduction

The interaction between the implant surface and the living body begins soon after the placement of the biomaterial in the body, and it has always been a challenge to determine the optimal modification to accelerate the biologic events which lead to faster osseointegration [1–3]. 

Since it has been proven that moderately rough surfaces outperform the turned surfaces [4–8], recent research has focused on further modifications that could possibly increase the bioactivity of the implant [9]. Thus, some of the state-of-the-art research has shifted to chemically modify moderately rough surfaces, which have been indicated to generate synergetic effects [10, 11]. Furthermore, the surface energy is another important factor involved in the regulation of osteogenesis. It has been said that depending on the surface energy, the surface state can either be hydrophilic or hydrophobic [12]. The energy state of the implant depends on the type of biomaterial, the handling during manufacturing, the mode of cleaning, sterilization, and needless to say, the handling of the implant during surgical procedure [13, 14]. In general, when the surface is positively charged, the surface turns hydrophilic and some of the plasma proteins essential for the initial osteogenic interactions adsorb to hydrophilic surfaces [15–17]. It has been suggested that the charge of the implant surface can be altered by oxidization [18], chemical and topographical modification [19, 20], and by plasma treatment [3, 14]. 

Plasma treatment is an interesting method to modify the implant surface. Not only can this treatment alter the surface charge, but this treatment can also alter the chemistry and the topography [21–23]. Thermal plasma treatment has been traditionally used as a method to utilize hydroxyapatite coatings on implant surfaces (plasma spraying) [24, 25]. Another form of plasma treatment, the atmospheric pressure (cold) plasmas, has shown to alter the surface energy and the chemistry due to the generation of high concentration of reactive species that are generated [21, 22]. This has been reported to be beneficial for the enhancement of osteogenic responses, as Duske et al. reported that surfaces treated with atmospheric plasma significantly enhanced the wettability and improved the initial cellular interaction [23].

The application of atmospheric plasma is increasing in numerous situations especially in the biomedical field due to their practical capability to low temperature providing plasmas that are not spatially bound or confined by electrodes [26, 27]. Moreover, this efficient and cost-effective process presents a potential benefit to any commercially available implant surface and has shown positive host-to-implant response when implants were plasma treated immediately prior to placement in the surgical sites [3]. While promising results have been achieved by the atmospheric treatment of endosseous implants prior to placement, it is also of interest to evaluate whether such surface modification is effective over longer periods of time, since the surface may be contaminated when the implant is reexposed to air [14, 28]. Stachowski et al. has reported that there is a possibility to maintain the high surface energy state of the titanium implant for at least 30 days, depending on various factors such as storage conditions [29]. The reason for 30 days storage of plasma-treated implants is to simulate a scenario of large-scale production by dental implants manufacturers, where the storage after surface modification may occur for several days prior to reaching the dental practitioner.

Thus, the objective of the present study was to investigate whether the biologic effect of an argon-based nonthermal plasma-treated dental implant surface stored for 30 days before the placement is still effective in terms of surface charge as compared to its untreated counterpart.

## 2. Materials and Methods

This study utilized 3.75 mm in diameter by 10 mm length nonwashed resorbable blasting media surface implants (Touareg with Osseofix Surface, Adin Dental Implants Systems Ltd., Afula, Israel). Half of the samples utilized were plasma treated 30 days prior to implantation (20 implants; experimental group), and the other half were placed as provided by the manufacturer (20 implants; control group). In summary, the control surface is fabricated by grit-blasting the surface with a proprietary bioactive ceramic powder prior to cleaning and sterilization, resulting in a textured surface with amounts of Ca and P close to 10% of the implant surface area.

The plasma was applied with a KinPen device (length = 155 mm, diameter = 20 mm, weight = 170 g) (INP-GreifSwald, Germany). The KinPen was used for the generation of a plasma jet at atmospheric pressure connected to a high-frequency power supply (1.5 MHz, 2–6 kV peak-to-peak, system power 230 V, 65 W), and the gas supply unit was connected to a gas controller (Multi Gas Controller 647C, MKS Instruments, Andover, MA). Argon tanks were attached to the gas controller with gas flow set at 5 standard liters per minute (slm). The plasma-treated implants were stored in their original vials before surgery for a period of thirty days. 

Six implants of each treatment (plasma 30 days prior to placement, plasma immediately prior to surface characterization, and control) were referred to physicochemical characterization. The surface morphology was observed by scanning electron microscopy (SEM, Philips XL 30, Eindhoven, The Netherlands) at ×5000 magnification and an acceleration voltage of 20 kV (*n* = 3 per surface).

In order to assess the surface energy of the surfaces, the Owens-Wendt-Rabel-Kaelble method was utilized [30]. For this purpose, 500 *μ*L droplets of distilled water, ethylene glycol, and diiodomethane were deposited on the surface of each implant group with a micropipette (OCA 30, Data Physics Instruments GmbH, Filderstadt, Germany). Images were captured and analyzed using software (SCA30, version 3.4.6 build 79). The relationship between the contact angle and surface energy was determined and was calculated by *γ*_*L*_ = *γ*_*L*_^*D*^ + *γ*_*L*_^*P*^, where *γ*_*L*_ is the surface energy, *γ*_*L*_^*D*^ is the disperse component, and *γ*_*L*_^*P*^ is the polar component.

Surface-specific chemical assessment was performed by X-ray photoelectron spectroscopy (XPS). The implants (*n* = 3, each group) were inserted in a vacuum transfer chamber and degassed to 10^−7^ torr. The samples were then transferred under vacuum to a Kratos Axis 165 multitechnique XPS spectrometer (Kratos Analytical, Chestnut Ridge, NY). Survey and high-resolution spectra were obtained using a 165 mm mean radius concentric hemispherical analyzer operated at constant pass energy of 160 eV for survey and 80 eV for high resolution scans. The take-off angle was 90°, and a spot size of 150 *μ*m × 150 *μ*m was used. The implant surfaces were evaluated at various locations.

Five male adult beagle dogs (approximately 1.5 years of age) were used for the study under approval of the bioethics committee for animal experimentation (CEUA 172/11) at the Universidade Federal de Uberlandia, Brazil. The pre anesthetic procedure comprised an intramuscular administration of atropine sulfate (0.044 mg/Kg) and xylazine chlorate (8 mg/Kg). General anaesthesia was then obtained following an intramuscular injection of ketamine chlorate (15 mg/Kg). Surgical procedures for bone access and wound closure have been described in detail elsewhere [31, 32].

The different implant surfaces were alternately placed from proximal to distal at distances of 1 cm from each other along the central region of the bone, and the start surface site (control and experimental) was alternated between animals. The implant distribution resulted in an equal number of implants for the 2-week comparison for both surfaces.

Postsurgical medication included antibiotics (penicillin, 20.000 UI/Kg) and analgesics (ketoprofen, 1 mL/5 Kg) for a period of 48 hours postoperatively. The animals were euthanized after a postsurgical period of 2 weeks by anesthesia overdose and the tibiae were retrieved by sharp dissection. Half of the implants were removal torqued and the other half were referred to nondecalcified histology processing as reported previously.

Histomorphometric analyses were carried out for each implant with the measurement of bone-to-implant contact (BIC) and bone area fraction occupancy (BAFO). The bone-to-implant contact (BIC) was determined at 50X–200X magnification (Leica DM2500 M, Leica Microsystems GmbH, Wetzlar, Germany) by means of computer software (Leica 8 Application Suite, Leica Microsystems GmbH, Wetzlar, Germany). The regions of bone-to-implant contact along the implant perimeter were subtracted from the total implant perimeter, and calculations were performed to determine the BIC percentage. The bone area fraction occupancy (BAFO) between threads in both cortical and trabecular bone regions was determined at 100X magnification (Leica DM2500 M, Leica Microsystems GmbH, Wetzlar, Germany) by means of computer software (Leica Application Suite, Leica Microsystems GmbH, Wetzlar, Germany). The areas occupied by bone were subtracted from the total area between threads, and calculations were performed to determine the BAFO (reported in percentage values of bone area fraction occupancy) [33].

Following the data normality check, statistical analysis was performed by paired  *t*-tests at 95% level of significance.

## 3. Results

**Figure 1:**
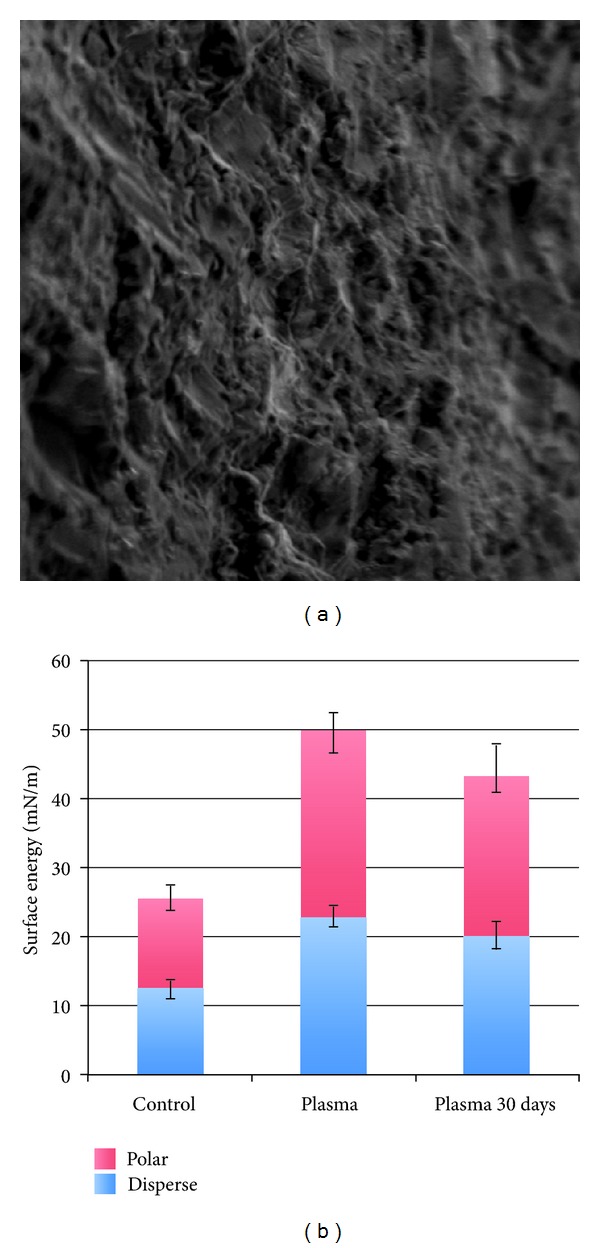
(a) Scanning electron microscopy micrograph (1000X) of the NWRBM implant surface and (b) surface energy measurements of the different groups (mean ± SD).

The scanning electron micrographs of the implant surface revealed a textured microstructure (Figure 1(a)). The surface energy assessment showed a substantial increase in both polar and disperses components immediately after plasma treatment and a slight decrease in both components 30 days after plasma treatment. Relative to untreated surfaces (control), the 30-day plasma-treated surfaces (experimental) presented higher polar and disperse components and an overall higher surface energy (Figure 1(b)).

The XPS analysis showed peaks of Ti, V, Al, C, Ca, O, and P for both groups tested. The control surface presented atomic percent values of 32.9, 9.8, 41.3, and 8.3 for C, Ca, O, and P, respectively, while the surface analyzed immediately after plasma treatment presented atomic percent values of 15.3, 12.2, 50.3 and 9.3 for C, Ca, O, and P, respectively. Relative to the control surface, the experimental surface presented increases in Ca, O, and P atomic percent levels at 10.4, 46.8, and 8.4, respectively, in addition to a decrease in C content at 24.6 atomic percent (Table 1).

**Figure 2:**
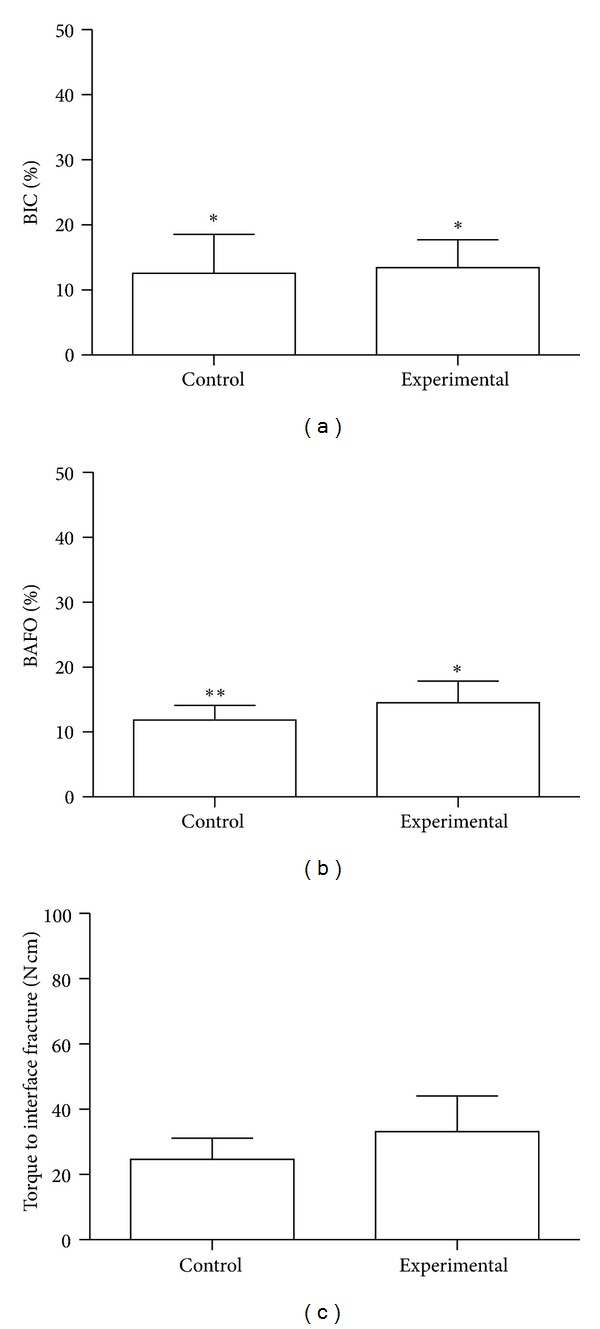
(a) Bone-to-implant contact (BIC), (b) bone area fraction occupancy (BAFO) percentages, and (c) raw torque data (mean ± 95% CI) for the control and experimental groups in the experimental period. The number of asterisks depicts statistically homogeneous groups.

No complications during animal surgical procedures and followup were observed, and all implants were clinically stable immediately after euthanasia. While no significant difference was observed for BIC parameter (*P* > 0.75), significantly higher levels of BAFO (*P* < 0.01) and torque (*P* < 0.02) were observed for the experimental group (Figures 2(a)–2(c)). 

**Figure 3:**
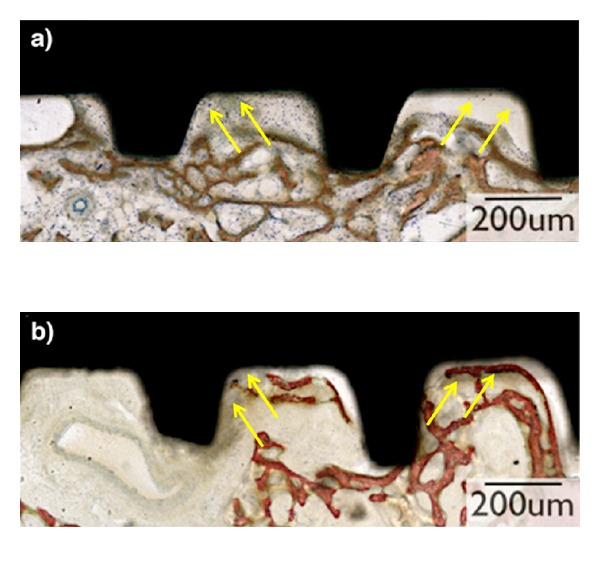
Representative overview of the histological micrographs of the plateaus at 2-week experimental period. (a) The histologic sections of the NWRBM group although presented layers of early connective tissue (stroma) filling the region between plateaus (arrows), there are some areas that the stroma collapsed (arrows). (b) The histologic sections of the Plasma 30 days group showed initial signs of bone formation adjacent to the implant surface (arrows) and the presence of layers of early connective tissue (stroma) filling the region between plateaus without detachment of the surface (arrows).

The histologic sections of the experimental group showed initial bone formation adjacent to the implant surface and the presence of layers of early connective tissue filling the region threads in a more intimate fashion than the control implants (Figure 3). In addition, the bone filled the region between implant threads in proximity to the implant inner diameter for the experimental group. Such observation could not be identified for the control group, where the bone formed distant from the implant inner diameter and the osteogenic connective tissue was not in as intimate contact with the implant surface as the experimental group (Figure 3).

## 4. Discussion

Previous SEM and optical interferometry assessment showed that the roughness of the utilized in the present study was similar to that of several other commercially available products [1, 34]. From a surface chemistry standpoint, the nonwashed resorbable blasting media treatment resulted in Ca and P comprising close to 10% of the surface elemental chemistry.

The surface energy assessment after Ar-based nonthermal plasma (NTP) application showed a substantial increase in surface energy (in both polar and disperse components) for the implants immediately after plasma treatment and that such increase was slightly lost 30 days after treatment. The disperse component of the surface energy characterizes the interaction between the surface and the dispensed liquid in terms of the nonpolar interactions between molecules. The roughness, unevenness, and the branching level of the surface determine this component. The polar component of the surface energy characterizes the polar interaction between the surface of the material and the working fluid. This component is determined by the presence of polar groups, electric charges, and free radicals on the surface [35].

The XPS results showed that surface elemental chemistry was modified by the Ar-based NTP treatment and that this change resulted in a higher degree of exposure of the surface chemical elements mainly at the expense of the removal of adsorbed C species immediately after plasma treatment [34]. Such surface exposure also slightly decreased as a function of time after plasma treatment as the 30-day plasma-treated group (experimental) presented elemental chemistry and showed evidence of adsorbed carbon species on the surface relative to implants evaluated immediately after plasma treatment. Nonetheless, relative to the control group a higher amount of surface exposure was still detected and was likely related to the removal of the adsorbed C species from the surface. Overall, both surface energy and XPS results supported that the plasma treatment presented potential of changing bone healing kinetics after placement 30 days after argon plasma treatment as surface energy and chemistry were still altered relative to the control group, suggesting that the effect of the plasma treatment was still effective after 30 days of storage.

Unlike our previous studies where the KinPen device was utilized immediately prior to implant placement, the present study considered that the device may not be readily available to all clinicians but utilized by implant manufacturers several days before the implant is placed. Thus, the present investigation is the first of a series of studies necessary to support the application of plasma on implants surface and prove the maintenance of their chemical properties over short and long periods of storage. 

The histologic study suggested that intimate interaction between tissues and implant surface occurred for the experimental group relative to the control. It is probable that more intimate relationship between the collagen fibers in the bone and implant surface resulted in the significantly higher torque and BAFO results detected for the experimental group.

These results obtained in the present study are in agreement with previous work that showed that surface wettability is beneficial in hastening osseointegration at early times *in vivo* [15, 36–39]. It has been demonstrated that increasing the surface energy of a grit-blasted implant surface by means of proprietary cleaning and storage in isotonic solution hastened osseointegration of dental implants at early implantation times relative to controls presenting the same surface roughness profile but lower surface energy levels [15].

In contrast to NTP treatment, where any given implant surface may be treated immediately prior to placement, the implant is stored in isotonic solution, so that the gain in surface energy is maintained. In contrast to this scenario, NTPs applied immediately prior to implantation has shown to be effective in altering the surface energy and chemistry resulting in a hastened host-to-implant response; however, concerns related to NTPs potential shelf life has been raised [3, 37, 40].

The present study partially answers the question as to whether NTPs present adequate shelf life for potential manufacturing based surface treatment, and further studies concerning longer periods of time are warranted. It is acknowledged that the main limitation of the present study is the absence of implants treated with plasma immediately prior to implantation, and such limitation impaired the evaluation of relative changes in bone response to NTP treated implants stored for 30 days in comparison to its treated and immediately placed counterpart.

## 5. Conclusion

Our results demonstrated that the surface elemental chemistry was modified by the plasma and lasted for 30 days after treatment, resulting in improved biomechanical fixation and bone formation at shortly after implantation compared to the control group.

## Figures and Tables

**Table 1:** X-ray photoelectron spectroscopy (XPS) spectra for both NWRBM, immediately treated plasma (Plasma), and Plasma 30 days surfaces (mean ± SD).

Chemical element (%)	NWRBM	Plasma	Plasma 30 days
Al2p	1.04 (0.2)	3.94 (1.2)	2.8 (1.5)
C1s	32.91 (2.1)	15.25 (1.6)	24.6 (3.3)
Ca2p	9.84 (1.1)	12.2 (2.1)	10.4 (2.4)
o1s	41.27 (3.2)	50.3 (3.7)	46.8 (5.2)
P2p	8.28 (0.8)	9.3 (1.6)	8.4 (2.7)
Ti2p	3.01 (0.4)	5.2 (1.4)	4.6 (2.3)
V2p3	0.16 (0.2)	0.9 (0.5)	0.7 (0.5)
